# Splenic abscess due to non-operative management of splenic injury: a case report

**DOI:** 10.1186/s13256-023-04026-5

**Published:** 2023-07-16

**Authors:** Zahra Moghimi, Ehsan Sadeghian, Aidin Yaghoobi Notash, Ehsan Sobhanian

**Affiliations:** 1 Department of Gynecology, Yas Hospital, Tehran University of Medical Sciences, Tehran, Iran; 2 Department of Surgery, Shariati Hospital, Tehran University of Medical Sciences, Tehran, Iran; 3 Tehran University of Medical Sciences, Tehran, Iran; 4 Department of Surgery, Sina Hospital, Tehran University of Medical Sciences, Tehran, Iran

**Keywords:** Splenic abscess, Splenic injury, Diabetes, Splenectomy

## Abstract

**Background:**

Splenic abscess is a rare disease, with incidence of 0.2–0.7% in previous studies. It often appears with left upper quadrant abdominal pain, fever, chills. Splenic abscess often happens because of hematogenous spreading of infections, endocarditis, angioembolization and some other rare reasons. Treatment relies on one of these two methods: percutaneous drainage or surgery.

**Case presentation:**

A 68-year-old diabetic Asian female (Asian woman) presented with generalized abdominal pain, low blood pressure, tachycardia, fever, lethargy and elevated level of blood sugar. She had history of conservative therapy in intensive care unit due to blunt abdominal trauma and splenic injury. She had a huge splenic abscess in ultrasonography and computed tomography scan so she went under splenectomy. Our patient had a splenic abscess without performing any intervention like angioembolization.

**Conclusion:**

Immune compromised patients who are selected for nonoperative management after splenic injury need close follow up and evaluating about abscess formation for at least 2 weeks. Early diagnosis and treatment with two methods including percutaneous drainage or splenectomy should be considered and it depends on patient’s risk factors, vital signs, general conditions and presence or absence of sepsis.

## Background

Splenic abscess is a rare disease, with incidence of 0.2–0.7% in previous studies [1] splenic abscess often appears with left upper quadrant abdominal pain, fever, chills and patients have leukocytosis and pleural effusion [2] The most cause of splenic abscess is hematogenous infection spreading, preceding trauma or other mechanisms [3–5], Endocarditis [6] and with a rare incidence pancreatic disease are known as a splenic abscess reason [7]. Diagnosis of splenic abscesses has become more frequent and faster in these years due to increasing number of immunocompromised patients who are probably at risk for this disease also prognose in this group is not good, and the extensive use of diagnostic imaging modalities includes ultrasonography (US) and computed tomography (CT) [8–11]. *E-coli*, *Streptococcus*, *Staphylococcus*, *Enterococcus*, *Klebsiella pneumoniae* are the most common cause in cultures [11]. Treatment relies on one of these two methods: percutaneous drainage or surgery [12, 13] However, due to the high failure rate of percutaneous drainage, splenectomy often considered as a final treatment for splenic abscesses [14] splenic abscess after angioembolization is also reported in some studies [15–17].

## Case presentation

A 68-year-old Asian female (Asian woman) presented with generalized abdominal pain since 5 days before admission, 80/50 mmHg blood pressure, 110 beats per minute, 39 centigrade degree of body temperature, lethargy and blood sugar = 634 mg/dl. She had soft abdomen in palpation and generalized tenderness. She had a history of blunt abdominal trauma 2 weeks ago and was admitted in intensive care unit in another center for one week, and she was under nonoperative management for splenic sub capsular hematoma (grade 3). The patient was not a suitable candidate for splenectomy due to stable vital signs and the grade of spleen injury and some risk factors as ischemic heart disease, diabetes mellitus, ejection fraction ≤ 35%, but negative past surgical history. She had a history of arbitrarily outpatient oral antibiotic therapy (Levofloxacin) due to fever during last week.

### Investigations

The laboratory findings revealed metabolic acidosis, 12.8 × 10^3^/L leukocytosis and neutrophilia of 97% and anemia (Hemoglobin = 7.6), blood sugar: 634 mg/dl, creatinine: 1.9 mg/dl, CRP = 245 mg/L.

**Fig. 1:**
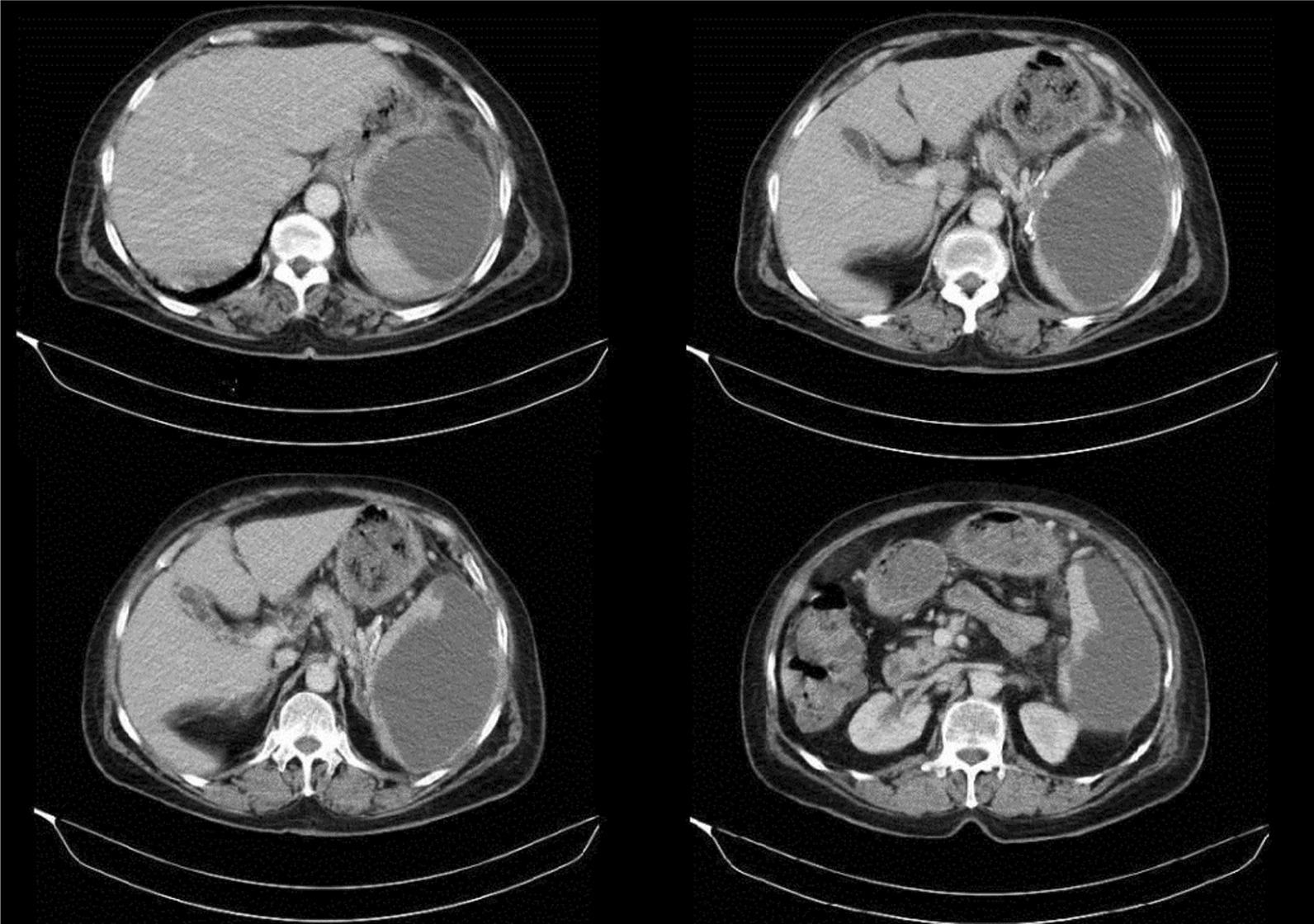
CT (computed tomography) scan showing a large splenic abscess measuring 10.8 × 8.7 × 5.3 cm

Initial clinical assessment in the admission unit revealed splenomegaly and a huge abscess that contains with 800 cc liquid in US and CT scan also confirmed these data and showed a large splenic abscess measuring 10.8 × 8.7 × 5.3 cm (centimeters) (Fig. 1).

### Treatment

**Fig. 2:**
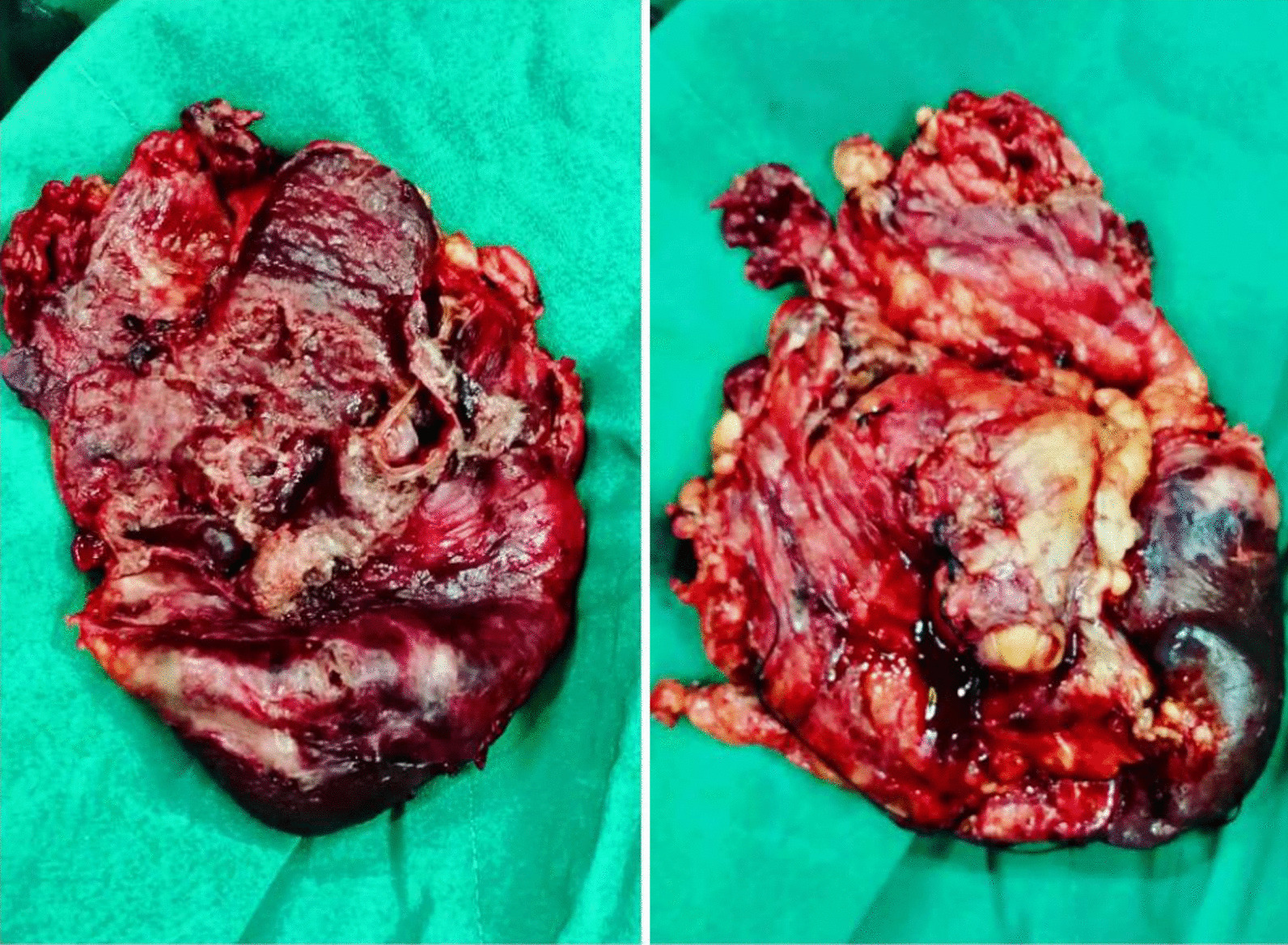
Spleen tissue with necrotic wall and ruptured capsule

**Fig. 3:**
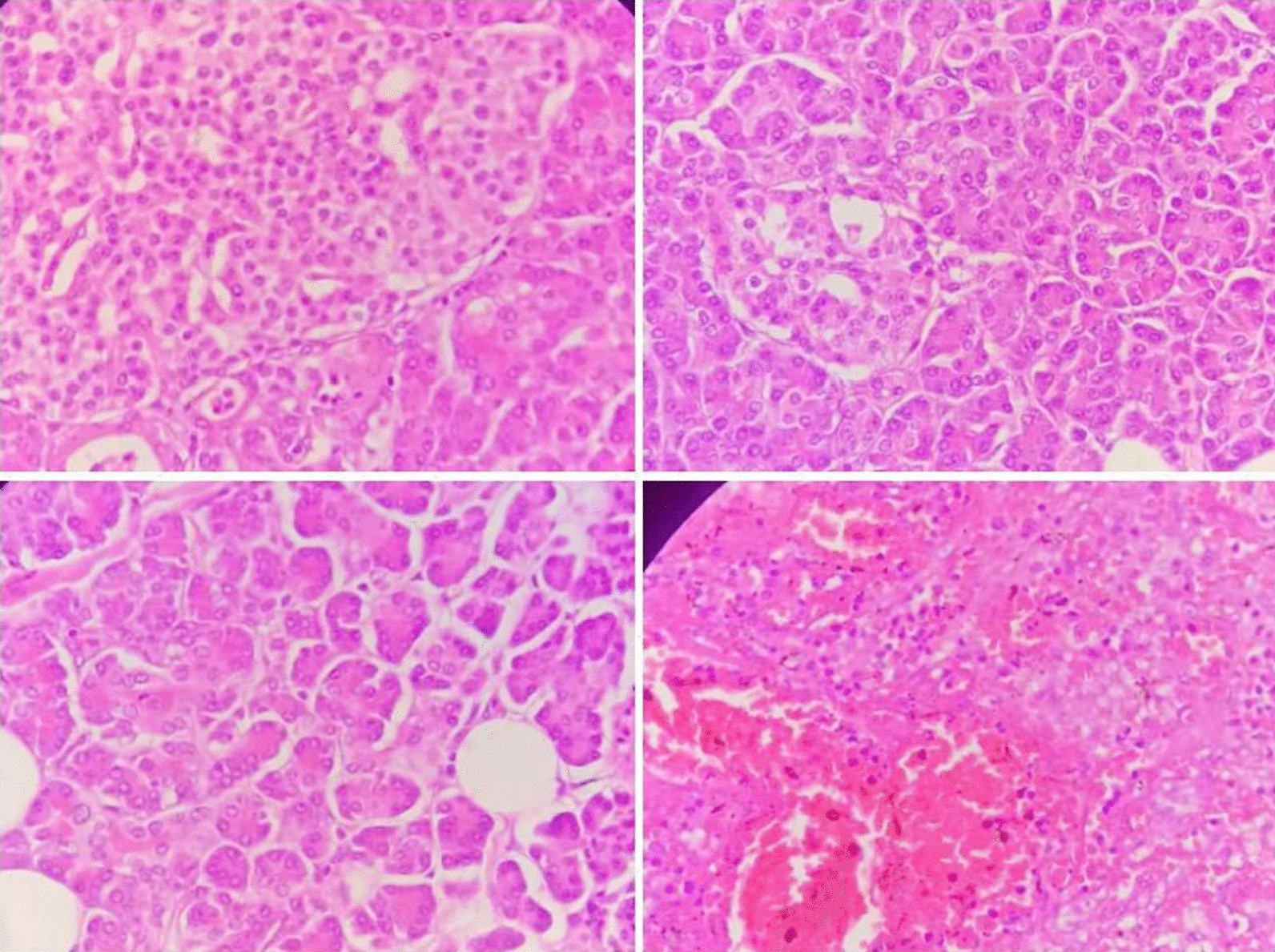
A neutrophilic infiltrate may be seen subcapsularly, intraparenchymal hemorrhage, massive congestion, diffuse immunoblastic and plasmacytic proliferation, and outpouring of neutrophils in the red pulp are also evident

The patient underwent resuscitation before surgery with 1 L intravenous 0.9% sodium chloride saline and 2 units packed red blood cell and prepared for operating room. Surgery started with a midline incision, during surgery sever inflammation and capsular adhesion to colon, gastric and abdominal wall were the most important points. Splenic wall was necrotic and it ruptured immediately after touching, and 1.5 L of creamy and white and malodor pus poured out from splenic abscess. Other organs were intact. Finally, splenectomy was performed and samples sent to the pathology and cytology (Figs. 2, 3). We inserted a drain and the secretions from that were clear (serous secretion) and dried out after 3 days.

### Outcome and follow-up

The patient was admitted in ICU after surgery and was treated by intravenous insulin drip and received intravenous antibiotics (Meropenem 500 mgr/TDS, Vancomycin 1 gr/BID) The remarkable point was that Blood culture, urine culture and even the culture of pus from the abscess were negative. Blood sugar levels and DKA (diabetic ketoacidosis) were controlled during hospitalization and biochemical inflammatory markers improved considerably and were in normal range before the patient’s discharge from the hospital. (CRP = 22 mg/dl, WBC = 8000/L) After one week taking care of patient in ICU the patient was transferred to surgery ward and stayed there for 5 days then she was discharged with good general condition.

## Discussion

A splenic abscess is a rare disease with frequency of 0.2–0.7% in autopsy series [1]. Left upper quadrant abdominal pain, fever and splenomegaly is the classical presentation. Imaging includes US and CT scan is necessary to diagnosis. Diabetes mellitus was the main underlying disease in Kuo-Chin chang *et al.* study in 2016 an article with 67 cases of splenic abscess during 18 years (1986–2004) witch shows the rarity of splenic abscess [11]. Nonoperative management of splenic injury is depending upon the grade of splenic injury, patient hemodynamic stability, absence or presence of other organ injuries and medical comorbidities [15–17]. Nonoperative management includes observation and embolization is used to manage 50–70% of patients with low grade injuries (I, II, III) [18–21]. Our patient was a nonoperative management candidate because she had a stable hemodynamic and she didn’t need to blood transfusion, her splenic injury was low grade and she had many medical comorbidities.

There are 3 choices for removing splenic pus collection: 1. Drainage (tap-nonsurgical) 2. Excision (open surgery) 3. Splenectomy. Patient clinical status, abscess location, size of the abscess, number of the abscess (single or multiple) and the local expertise are important factors for decision and its individualized [22].

Our patient was a surgical candidate due to huge abscess size, septic shock, abdominal examination (acute abdomen) and possibility of wall necrosis that drainage would cause abscess rupture and peritonitis. Besides we didn’t had the facility of percutaneous drainage in our center.

Also angioembolization did not perform in this case. Splenic abscess has been reported in some studies in patients who were treated with angioembolization conservative therapy [15–17]. Our patient had a splenic abscess without performing any intervention. The patient had immune insufficiency due to diabetes mellitus, all of the cultures were negative similar to Tartagli *et al. *study [17]. Negative cultures might be related to her recent antibiotic therapy (Levofloxacin) and also can be a lab data bios of our center too.

Intraabdominal and peri splenic abscess is reported after nonoperative management in Michel Paul Johan Teuben *et al.* study and no splenic abscess reported. The median length of hospital stay in this study were 13 days [23]. while our patient hospitalized overall for 3 weeks.

Some studies recommend surgical treatment in patients older than 55 year due to higher failure rate of nonoperative management [24–27] while some other studies believe age should not consider in patient management [28, 29].

## Conclusion

It seems to choosing between conservative therapy versus surgery in splenic injury is individualized. The most important issue is to select suitable patient and consider the risk of splenic abscess. Because performing splenectomy due to splenic abscess is accompanied by great difficulty. Patients who are candidate for nonoperative management specially whom with immunodeficiency needs long term follow up (at least 2 weeks). Therefore we would recommend to consider abscess formation probability in high risk patients who are candidates for conservative therapy in splenic rupture.

We strongly recommend that patients with splenic abscess undergo surgery in referral centers due to the need for special care and facilities during and after surgery.

## Data Availability

Not applicable.

## Abbreviations

CT: Computed tomography
US: Ultra sound
CM: Centimeters
EF: Ejection fraction
